# A Novel Lipidomic Strategy Reveals Plasma Phospholipid Signatures Associated with Respiratory Disease Severity in Cystic Fibrosis Patients

**DOI:** 10.1371/journal.pone.0007735

**Published:** 2009-11-06

**Authors:** Ida Chiara Guerrera, Giuseppe Astarita, Jean-Philippe Jais, Dorota Sands, Anna Nowakowska, Julien Colas, Isabelle Sermet-Gaudelus, Martin Schuerenberg, Daniele Piomelli, Aleksander Edelman, Mario Ollero

**Affiliations:** 1 Plateau Proteome Necker, Université Paris Descartes, IFR94, Paris, France; 2 INSERM, U845, Université Paris Descartes, Faculté de Médecine, Paris, France; 3 University of California Irvine, Irvine, California, United States of America; 4 Université Paris Descartes, Service de Biostastistiques et Bioinformatique du CHU Necker-Enfants-Malades, Paris, France; 5 Institute of Mother and Child, Warsaw, Poland; 6 Bruker Daltonics, Bremen, Germany; University of Alabama-Birmingham, United States of America

## Abstract

The aim of this study was to search for lipid signatures in blood plasma from cystic fibrosis (CF) patients using a novel MALDI-TOF-ClinProTools™ strategy, initially developed for protein analysis, and thin layer chromatography coupled to MALDI-TOF (TLC-MALDI). Samples from 33 CF patients and 18 healthy children were subjected to organic extraction and column chromatography separation of lipid classes. Extracts were analyzed by MALDI-TOF, ion signatures were compared by the ClinProTools™ software and by parallel statistical analyses. Relevant peaks were identified by LC-MSn. The ensemble of analyses provided 11 and 4 peaks differentially displayed in CF *vs* healthy and in mild *vs* severe patients respectively. Ten ions were significantly decreased in all patients, corresponding to 4 lysophosphatidylcholine (18∶0, 18∶2, 20∶3, and 20∶5) and 6 phosphatidylcholine (36∶5, O-38∶0, 38∶4, 38∶5, 38∶6, and P-40∶1) species. One sphingolipid, SM(d18∶0), was significantly increased in all patients. Four PC forms (36∶3, 36∶5, 38∶5, and 38∶6) were consistently downregulated in severe *vs* mild patients. These observations were confirmed by TLC-MALDI. These results suggest that plasma phospholipid signatures may be able to discriminate mild and severe forms of CF, and show for the first time MALDI-TOF-ClinProTools™ as a suitable methodology for the search of lipid markers in CF.

## Introduction

Cystic fibrosis (CF) is characterized by a vicious circle of chronic inflammation in pulmonary airways and recurrent exacerbations, usually concomitant with bacterial infections [1]–[3]. These manifestations account for the most part of morbidity and mortality associated with the disease. CF is attributed to mutations in the *CFTR* gene, coding for the Cystic Fibrosis Transmembrane Regulator, a multifunctional transmembrane chloride channel [4]. Nevertheless there is no direct correlation between the presence of a particular mutation and the severity status [5]. A significant improvement of clinical parameters, such as FEV1, and of life expectancy has been obtained in the last decades. Nearly twenty products are currently in the clinical trial pipeline, covering channel function repair, rescue of CFTR protein, gene therapy, antibacterial and anti-inflammatory strategies, as reported by the Cystic Fibrosis Foundation (http://www.cff.org/treatments/Pipeline/). The choice of a particular strategy should be made depending on individual characteristics of patients. This prompts the need of their phenotypical characterization based on the search for markers that could predict the evolution of the disease. An early marker of exacerbations or a prognostic indicator could be extremely helpful in establishing the most adapted treatment for each particular case, aiming at limiting lung damage. At the beginning of the decade studies for protein biomarkers in body fluids by non-invasive methods were initiated. Proteins like myeloperoxidase, IL-8, cleaved alpha-antitrypsin, and S100A8 (or CF antigen), have been found in sputum as predictors of pulmonary exacerbations [6], or associated with the presence of CFTR mutations [7], [8], in addition to proteomic signatures in serum corresponding to inflammation markers [9]. Other proteins differentially expressed in target tissues of CF before the appearance of any sign of the disease, like annexin-1 (also found cleaved in the sputum of CF patients) [10], cytosolic phospholipase A2α [10], cytokeratins 8 and 18 [11], peroxiredoxin 6 [12] may also be part of a proteomic signature. More recently, mass spectrometry analysis of sputum has revealed a proline-glycine-proline peptide (PGP), an extracellular matrix-derived neutrophil attractant, as a marker of inflammatory exacerbation in CF [13]. Concerning lipids, although imbalances in fatty acid profiles [14]–[17], and other lipid moieties (reviewed in [18], [19]) in CF patients and models have been consistently reported, no systematic search by comprehensive lipidomic techniques has been performed to date.

The progress in mass spectrometry in the last years has democratized these techniques. They are now currently used to answer not only chemistry-related questions, but also in biology and the medical field. They allow the detection, identification and quantification of less abundant molecules important in cell signaling and homeostasis. Matrix-Assisted Laser Desorption Ionization Time of Flight (MALDI-TOF) mass spectrometry (MS) has proven effective in the analysis of most lipid classes [20]. However it has not been extensively utilized for lipid analysis as compared to other ionization methods, such as electrospray (ESI) or electron impact (EI). MALDI presents several advantages with respect to other mass spectrometry approaches, such as rapidness and reproducibility, which makes it especially suitable in studies involving a large number of samples. A novel application of MALDI, coupling thin layer chromatography and mass spectrometry (TLC-MALDI) reveals as an interesting alternative when searching for specific differences in individual lipid molecules. This technology has recently been developed and applied to the analysis of lipids in stem cells [21].

In the present study we use for the first time a combination of MALDI-TOF, TLC-MALDI and ESI-MS/MS on plasma samples from CF patients and control individuals in the search for lipid signatures associated with the presence of CFTR mutations and with the severity of the respiratory disease characteristic of CF.

## Methods

### Ethics Statement

All protocols were approved by the Komisja Bioetyczna przy Instytucie Matki i Dziecka w Warszawie (ethical committee, Institute for Mother and Child, Warsaw, Poland). All patients involved in the study or their parents or legal guardians signed a written consent form.

### Patient Description and Sample Collection

**Figure 1:**
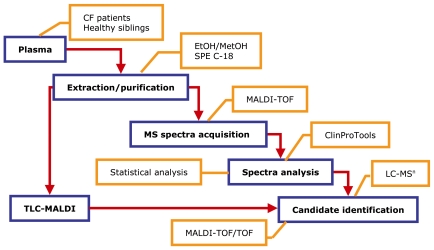
Flow diagram describing the experimental procedures.

Blood plasma samples were collected from CF patients and their relatives (including healthy siblings) at the Institute of Mother and Child (Warsaw, Poland) using a standard clinical protocol. Patients were categorized into mild (no more than 2 pulmonary exacerbations a year requiring intravenous antibiotics) and severe (more than 2 pulmonary exacerbations a year requiring intravenous antibiotics, persistent inflammatory changes in lungs, bronchiectasis, hemoptisis, hemorragia). All patients were at a good nutritional status, and out of exacerbation period at the time of collection. All pancreatic sufficient patients at the time of the study developed pancreatic insufficiency at a later stage. The analysis was performed on samples from 18 healthy, 33 mild, and 10 severe individuals (Patients' description is presented in Table 1). Sample collection was carried out with appropriate ethical committee approval. Samples were collected in VACUETTE® EDTA tubes K3E/EDTA K3 (Greiner Bio-One, Kremsmünster, Austria) and centrifuged at 2800 g for 15 min at 4°C. Plasma was separated and dispensed into 200-µl aliquots so that each aliquot was subjected to a single freezing-thawing cycle. Plasma samples were frozen in liquid nitrogen and stored at −80°C. The subsequent analytical procedure is schematically represented in Figure 1.

**Table 1:** Clinical data of CF patients (n.i.: not identified; n.d.: not determined).

code	gender	age at inclusion	tanner stage	genotype	pancreatic function	BMI	% ideal weight for height	pulmonary disease	FEV1%	Shwachman score
cf32	M	0,54	1	F508del/F508del	PI	12,3	69	severe	n.d.	70
cf38	M	0,55	1	F508del/F508del	PS	18,4	99,1	mild	n.d.	95
cf39	M	3,06	1	F508del/F508del	PI	15,3	93	mild	n.d.	85
cf42	F	11,14	1	F508del/F508del	PI	16,9	95,7	severe	76	65
cf44	F	7,11	1	F508del/F508del	PI	17,9	109	mild	113	85
cf47	M	13,57	3	F508del/F508del	PI	16,9	92	severe	121	65
cf48	M	10,69	1	F508del/F508del	PI	15,2	91	severe	85	65
cf51	M	1,05	1	F508del/F508del	PI	13,7	76,8	severe	n.d.	60
cf52	M	4,02	1	F508del/IVS8-ST	PS	12,8	81	mild	n.d.	75
cf60	M	6,26	1	F508del/F508del	PI	17	119	mild	116	90
cf64	F	5,14	1	F508del/F508del	PI	15	100	mild	n.d.	80
cf65	F	1,79	1	F508del/F508del	PI	15	88,1	mild	n.d.	75
cf69	M	7,23	1	F508del/F508del	PI	16,1	101,6	mild	94	90
cf70	F	5,07	1	F508del/F508del	PI	14,5	97,3	severe	n.d.	80
cf73	F	18,86	5	F508del/N1303K	PI	20	97,4	severe	100	65
cf74	M	9,70	1	F508del/N1303K	PS	17,2	97,7	mild	121	75
cf79	M	7,37	1	F508del/F508del	PI	14,5	100	severe	n.d.	80
cf93	M	17,07	5	F508del/F508del	PI	23,4	102	mild	87	90
cf96	M	10,48	1	F508del/n.i.	PI	14	92,6	mild	89	70
cf97	M	10,48	1	F508del/n.i.	PI	13,7	91,1	mild	86	70
cf104	F	23,48	5	F508del/2184ins	PI	21,05	n.d.	mild	110	90
cf106	F	17,74	4	F508del/2184ins	PI	14,4	71,1	severe	23,3	40
cf110	F	2,08	1	F508del/2143d/T	PI	16,6	106	mild	n.d.	80
cf114	F	0,91	1	F508del/R347P	PI	15,5	90,6	mild	n.d.	85
cf123	F	16,45	4	F508del/F508del	PI	20,9	101	mild	90	80
cf124	F	17,58	1	F508del/F508del	PI	21,1	102	mild	110	90
cf127	M	11,82	1	3849+10kB/n.i.	PI	11,9	78	severe	37	65
cf136	M	2,14	1	F508del/n.i.	PI	14,8	93,3	mild	n.d.	75
cf142	F	4,57		F508del/F508del	PI	14,8	93	mild	n.d.	85
cf147	M	6,89	1	F508del/R1066C	PI	18,5	102	mild	n.d.	80
cf148	M	0,16	1	F508del/R1066C	PI	14,6	94	mild	n.d.	85
cf151	F	4,38	1	F508del/F508del	PI	15,2	98	mild	n.d.	90
cf152	F	2,41	1	F508del/F508del	PI	14,2	88,4	mild	n.d.	70

### Lipid Extraction and Separation

Aliquots of 100 µl were subjected to organic extraction and purification allowing the sequential enrichment of different groups of molecules. Organic extraction was performed by addition of 6 volumes of chloroform∶methanol (2∶1. v/v). The organic phase was loaded onto a LC-NH2 aminopropyl column (Supelco, Bellefonte, PA), and three fractions enriched respectively in neutral lipids (#1), free fatty acids (#2) and neutral phospholipids, i.e. phosphatidylcholine, phosphatidylethanolamine and sphingomyelin (#3), were sequentially eluted with chloroform∶isopropanol (2∶1. v/v), ethyl-ether∶acetic acid (98∶2. v/v) and methanol, respectively, according to a method described elsewhere [22].

### Mass Spectrometry Analysis

Extracts were subjected to MALDI-TOF MS in both negative and positive modes in order to obtain signatures characteristic of any of the groups of individuals. Organic extracts were evaporated to dryness, resuspended in an equal volume of chloroform∶methanol (2∶1. v/v) and loaded on the MALDI target in combination with 1 volume of 0.5 M dihydrobenzoic acid (DHB) in methanol with 0.1% trifluoroacetic acid as the matrix. Although this matrix is specific for positive ionization, data were acquired in both the positive and negative reflectron mode between 200 and 2000 m/z in an AutoFlex MALDI-TOF mass spectrometer (Bruker Daltonics), using a low molecular weight peptide mixture (Peptide Mix-5, LaserBioLabs, Sophia-Antipolis, France) for calibration. Two MS signatures (in the positive and negative modes) were obtained from each extract fraction.

### Signature Comparisons and Statistical Analyses

Signatures corresponding to the different patient and control groups were compared by the ClinProTools™ software (Bruker Daltonics). This tool recalibrates and normalizes all spectra to their own total ion count, performs a statistical calculation and provides the significant differences for each ion peak among the groups of individuals considered. Statistical significance of differential displayed peaks was determined by either Student T-Test for 2 groups, or by ANOVA, for more than 2 groups.

ClinProTools-independent statistical analyses were performed with the R system (v2.9) and Bioconductor software packages (V2.4) [23], [24]. Peak amplitudes were log-transformed to normalize distributions. Analysis of variance (ANOVA) with robust variance estimation of the three groups of subjects was performed with the LIMMA package [25] to identify peaks differentially displayed. P values were established after false discovery rate correction. Those peaks showing p<0.05 were considered to present statistically significant differences. Hierarchical clustering was performed after centering of peak expressions using the Ward Agglomeration Algorithm and a distance defined by the correlation coefficient for peaks and an euclidean distance for samples.

### Peak Identification by Liquid Chromatography – Mass Spectrometry (LC-MS^n^)

LC-MS^n^ was performed in an 1100-LC system equipped with an Ion Trap XCT (Agilent Technologies). Lipids were separated using a Poroshell 300 SB C18 column (2.1×75 mm inner diameter, 5 µm; Agilent Technologies, Wilmington, DE) maintained at 50°C. A linear gradient of methanol in water containing 5 mM ammonium acetate and 0.25% acetic acid (from 85% to 100% of methanol in 4 min) was applied at a flow rate of 1 ml/min. Detection was set in either the positive or the negative mode. N_2_ was used as drying gas at a flow rate of 12 l/min, with temperature of 350°C and nebulizer pressure of 60 p.s.i. Helium was used as the collision gas. Lipids were identified by comparison of their LC retention times and MSn fragmentation patterns with those of authentic standards as previously described [26], [27]. Detection and analysis were controlled by Agilent/Bruker Daltonics software version 5.2. Briefly, in positive ESI mode, PC species were detected as protonated molecular ions and MS^2^ yielded product ions deriving from the neutral loss of the trimethylammonium and the phosphocholine head group. Using ESI set in negative mode, PC species were detected as acetate adducts of the molecular ions. The MS^2^ fragmentation pattern of PC species is characterized by neutral loss of the acetate adduct of the N-methyl group. MS3 of the ion [M-H-CH3]^−^ yields the lysophospholipid with neutral loss of ketene in combination with the sn-1 and sn-2 carboxylate anions.

### Thin Layer Chromatography – MALDI (TLC-MALDI)

Results were verified by a second approach (Figure 1) using a recently developed technology combining chromatographic separation by High Performance Thin Layer Chromatography (TLC) and MALDI-TOF MS [21]. Organic extracts were spotted on Silica Gel 60 HPTLC plates (Merck), pre-developed in chloroform-methanol (1∶1, v/v) and separated sequentially by two mobile phases: chloroform-ethanol-triethylamine-water (30∶35∶35∶8, v/v/v/v) and hexane-ether (100∶4.5, v/v). DHB matrix (100 mg/ml) in 50% acetonitrile was spotted using an ImagePrep apparatus (Bruker Daltonics) and a MALDI-TOF spectra scan in the positive mode was obtained by the TLC-MALDI and FlexImaging software packages (Bruker Daltonics). Ions of interest were subjected to MALDI-TOF/TOF for structure determination and eventual identification.

## Results

After exclusion of matrix background and isotopic peaks, a total of 100 relevant ions were detected in the neutral lipid fraction, 55 in the fatty acid fraction, and 58 in the phospholipid fraction, in the positive mode analysis. In the negative mode, relevant ions (11 in total) were only detected in the free fatty acid fraction. Group comparisons by ClinProTools™ resulted in the detection of 18 ion peaks, all of them corresponding to the phospholipid fraction analyzed in positive mode, differentially displayed with p-values <0.05 (Table 2). Differences were established between CF (all patients) *vs* non-CF children (14 peaks) and between patients showing mild *vs* severe pulmonary disease (7 peaks). When mild and severe patient groups were established according to Shwachman Score values (cutoff  = 65), analogous results were obtained (not shown). No significant differences were found when individuals were grouped according to pancreatic sufficiency, gender or age.

**Table 2:** Peaks differentially displayed in either healthy controls *vs* CF patients (*) or in CF mild *vs* CF severe patients (**) with p<0.05 after T-test analysis.

*m/z*	*Δ(m/z)*	*Relative abundance*				*Identity*
		*non-CF*	*CF*	*CF mild*	*CF severe*	
482.49	0.13	0.05±0.08	0.3±0.49*	0.26±0.43	0.38±0.63	LPC(O-16∶0) or LPE(18∶0)
489.27	0.07	0.06±0.21	0.29±0.41*	0.27±0.44	0.33±0.35	SM(d18∶0)
520.31	0.02	2.99±3.13	0.7±1*	0.84±1.14	0.98±2.1	LPC(18∶2)
524.35	0.02	2.53±2.59	0.77±1.28*	0.97±1.44	0.61±1.24	LPC(18∶0)
542.3	0.02	2.18±1.62	0.81±0.98*	0.94±1.07	0.45±0.56	LPC(20∶5)
544.31	0.02	1.74±1.08	0.8±0.88*	0.96±0.96	0.51±0.53	LPC(20∶4)
546.33	0.02	1.28±0.97	0.58±0.62*	0.7±0.67	0.29±0.3	LPC(20∶3)
725.5	0.03	5.54±4.18	3.61±2.83	4.41±2.87	1.52±1.26**	SM(16∶0)
780.53	0.01	34.18±14.25	21.84±20.71*	26.31±22.72	10.44±3.84**	PC(36∶5)
782.52	0.02	27.14±7.67	21.74±15.82	25.86±16.86	12.38±5.15**	PC(36∶4)
784.54	0.02	21.07±6.07	17.68±12.01	21.28±12.12	9.77±6.07**	PC(36∶3)
786.59	0.01	10.64±9.01	4.63±4.34*	5.96±4.38	5.27±13.07	PC(36∶2)
804.54	0.13	7.1±4.13	4.12±3*	5±3.01	1.76±1.07**	PC(O-38∶0)
806.56	0.00	9.54±3.98	6.41±4.55*	7.92±4.43	2.89±1.94**	PC(38∶6)
808.58	0.00	18.88±7.49	12.45±8.82*	15.29±8.6	5.66±3.68**	PC(38∶5)
810.6	0.01	5.77±1.83	3.63±2.87*	4.55±2.77	1.95±2.51	PC(38∶4)
828.56	0.11	1.66±1.37	0.53±0.46*	0.59±0.51	0.32±0.22	PC(P-40∶1)
832.56	0.16	3.02±1.85	1.78±1.64	2.14±1.73	0.8±0.83**	PC(O-40∶0)

All peaks are [M+H]^+^ except 489.27 and 725.53, which correspond to [M+Na]^+^. # Theoretical identity (not confirmed by MS^n^).

**Figure 2:**
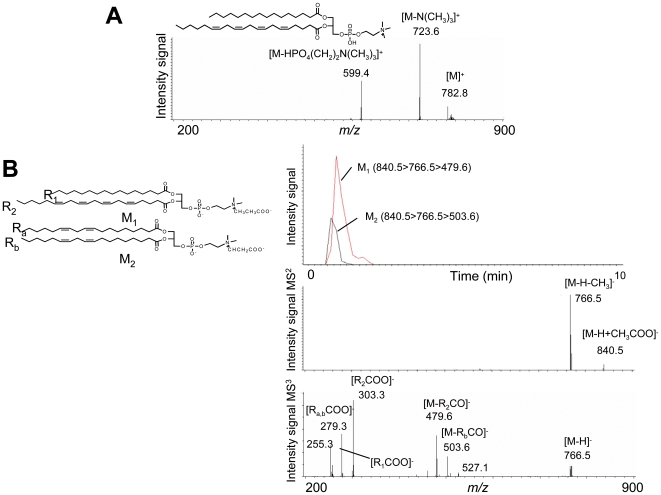
A: Representative LC/MSn analysis of 1-palmitoyl,2-arachidonoyl-phosphatidylcholine in human plasma sample. This PC species was detected as protonated molecular ion in the positive ion mode. MS2 yielded product ions deriving from the neutral loss of the trimethylammonium and the phosphocholine head group. B: Identification of 1-palmitoyl,2-arachidonoyl PC (*m/z* 840.5) in human plasma samples. LC/MS chromatogram (*upper panel*) and fragmentation pattern in MS2 and MS3 using an ion trap instrument (*upper and medium panels*). PC species were detected as acetate adducts of the molecular ions using ESI set in the negative mode. The MS2 fragmentation pattern is characterized by neutral loss of the acetate adduct of the N-methyl group (*medium panel*). MS3 of the ion with m/z 766.5 yields the lysophospholipid with neutral loss of ketene in combination with the sn−1 and sn−2 carboxylate anions (*lower panel*). Extraction of the LC/MS3 chromatogram allows to distinguish between different isomers (*upper panel*). Abbreviations: R1,a = sn−1 aliphatic chain; R2,b = sn−2 aliphatic chain.

Peaks of interest were subjected to LC-MS^n^ or MALDI-TOF/TOF for structure determination and, eventually, for identification. The identity of all the molecules was confirmed by MS/MS fragmentation (this procedure is summarized in Figure 2). This led to the unambiguous identification of 5 lysophosphatidylcholine (LPC), 10 phosphatidylcholine (PC), and 2 sphingomyelin (SM) species (Table 2) and one possible lysophosphatidylethanolamine (LPE). LPE(18∶0) and the sodium adduct of SM(d18∶0) (sphinganine-1-phosphocholine), were significantly increased in plasma from all CF patients. PC included two alkyl-acyl and one alkenyl-acyl (plasmalogen) form. In all cases the relative abundance of LPC and PC was decreased in all CF patients *vs* non-CF individuals. More interestingly, 7 of them, i.e. PC(36∶3), PC(36∶4), PC(36∶5), PC(O-38∶0), PC(38∶6) and PC(38∶5), as well as the sodium adduct of SM(16∶0) were also significantly decreased in severe as compared to mild CF patients.

**Figure 3:**
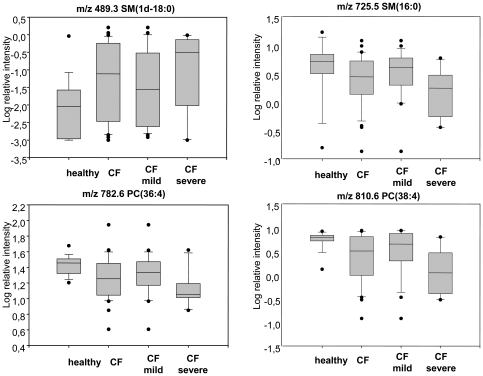
Box plots corresponding to four peaks differentially displayed. Two sphingomyelin (m/z 489.3 and 725.5, upper panels) and two phosphatidylcholine (m/z 782.6 and 810.6, lower panels) species are represented. Relative intensity corresponds to the area of each peak of the spectrum related to the total ion count. Box plots are constructed from log-transformed relative intensity data.

Representative examples of differences between all patients, healthy controls, mild and severe individuals are shown in Figure 3, which depicts box plots for 4 molecules after log transformation of raw data. The two lower panels correspond to PC forms (m/z 782.6 and 810.6) and the two upper to two SM (m/z 489.3 and m/z 725.5). As a general observation, LPC moieties (see Table I) were differentially displayed between all CF patients and healthy children, but not between the mild and severe forms of disease. Conversely, the differential presence of PC and SM species was significant between both mild and severe patients and between healthy and all sick individuals. The SM containing sphinganine as the sphingoid base (m/z 489.3) is exceptionally increased in all CF patients as compared to healthy controls.

**Figure 4:**
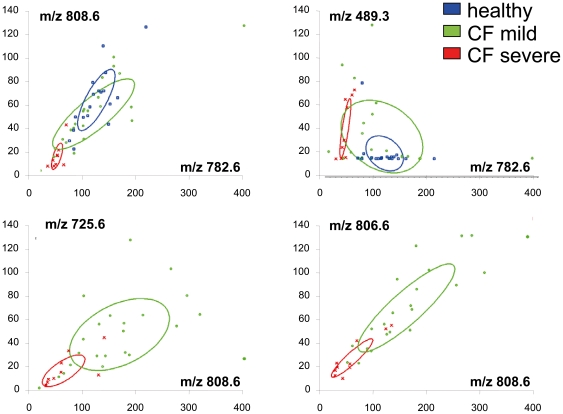
ClinProTools™ 2D Peak Distribution diagrams corresponding to four couples of ions. Ellipses correspond to 95% confidence intervals. Blue, green and red spots denote healthy subjects, mild patients and severe patients respectively.

The ability of differentially displayed peaks to segregate the populations of individuals considered is represented by 2D peak distribution diagrams (Figure 4). As shown in the plot, several combinations of peaks can separate healthy from severe individuals with a 95% confidence interval (i.e. 782.6/808.6 and 782.6/489.3, upper panels). Nevertheless, although there is a fairly good separation of mild and severe individuals, some overlapping occurs even if the best combination of peaks is chosen (806.6/808.6 or 725.5/808.6, lower panels).

**Figure 5:**
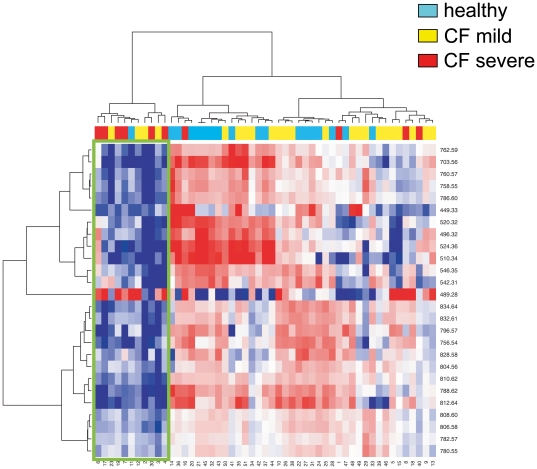
Hierarchical clustering heat map representing increases (red) and decreases (blue) in intensity of peaks significantly different in healthy *vs* CF patients. Peak masses are represented in the vertical axis, and individuals in the horizontal axis. Data were log-transformed, obtained after clustering of peaks (vertical axis) and individuals (horizontal axis), analyzed by the Ward Agglomeration Algorithm, and filtered for healthy *vs* CF patients comparison. The green trace delimits the data corresponding to the cluster of individuals that show consistent changes in lipid content.

**Figure 6:**
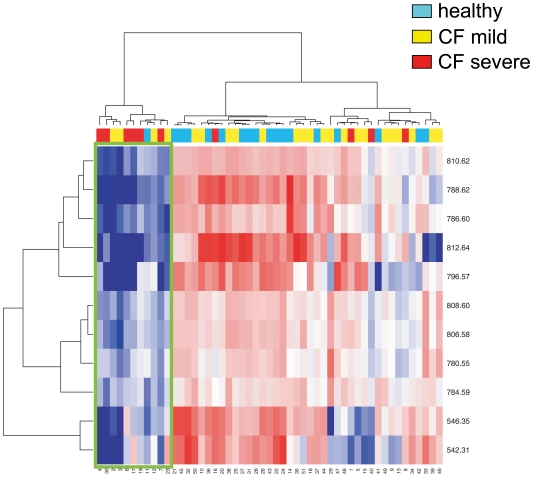
Hierarchical clustering heat map representing increases (red) and decreases (red) in intensity of peaks significantly different in mild *vs* severe patients. Peak masses are represented in the vertical axis, and individuals in the horizontal axis. Data were log-transformed, obtained after clustering of peaks (vertical axis) and individuals (horizontal axis), analyzed by the Ward Agglomeration Algorithm, and filtered for mild *vs* severe patients comparison. The green trace delimits the data corresponding to the cluster of individuals that show consistent changes in lipid content.

A parallel statistical analysis was performed by ANOVA after log transformation of raw data corresponding to the phospholipids fraction. The results are shown in Table 3. This study provided 26 peaks that changed significantly between healthy controls and all CF patients, and 11 peaks that changed between mild and severe patients. Concerning all CF *vs* nonCF evaluation, 11 peaks were coincident with the results of the ClinProTools™ analysis, corresponding to SM(d18∶0), LPC(18∶0), LPC(18∶2), LPC(20∶3), LPC(20∶5), PC(36∶5), PC(O-38∶0), PC(38∶4), PC(38∶5), PC(38∶6), and PC(O-40∶0). Concerning mild *vs* severe comparison, 4 peaks were coincident with those provided by ClinProTools™, namely PC(36∶3), PC(36∶5), PC(38∶5) and PC(38∶6). A hierarchical clustering analysis of these peaks is shown in two heat maps, after filtering according to either healthy *vs* all CF (Figure 5) or mild *vs* severe (Figure 6). In both figures, two major clusters of individuals can be observed: one represented on the left side, in which most of the peaks are downregulated (blue), constituted by most of the severe patients, and the right side, mostly characterized by predominant upregulation (red) of peaks. The peak corresponding to m/z 489.3 (sphinganine-1-phosphocholine) represents an exception, as its trend is opposite to the rest of peaks in most of the individuals (Figure 5).

**Table 3:** Peaks differentially displayed in either healthy healthy *vs* CF patients (a) or in CF mild *vs* CF severe patients (b) with p<0.05 after log transformation and ANOVA analysis.

*m/z*	*Δ(m/z)*	*Differential display*	*Identity*
449.33	0.03	a	MGL(22∶0)^#^
489.27	0.07	a	SM(d18∶0)^#^
498.32	0.04	a	PS(16∶0)^#^
510.34	0.05	a	LPC(O-18∶0)
520.31	0.02	a	LPC(18∶2)
524.35	0.02	a	LPC(18∶0)
542.3	0.02	ab	LPC(20∶5)
546.33	0.02	ab	LPC(20∶3)
703.58	0.01	a	SM(16∶0)
756.54	0.01	a	PC(34∶3)
758.55	0.02	a	PC(34∶2)
760.57	0.02	a	PC(34∶1)
762.59	0.01	a	PC(34∶0)
768.62	0.03	a	PE(38∶4)
780.53	0.01	ab	PC(36∶5)
782.52	0.02	a	PC(36∶4)
784,54	0.02	b	PC(36∶3)
786,59	0.01	b	PC(36∶2)
788.6	0.02	ab	PC(36∶1)
796.57	0.05	b	PC(O-38∶4) or PC(P-38∶3)^#^
798.57	0.07	a	PC(P-38∶2)
804.54	0.13	a	PC(O-38∶0)
806.56	0.00	ab	PC(38∶6)
808.58	0.00	ab	PC(38∶5)
810.6	0.01	ab	PC(38∶4)
812.64	0.02	ab	PC(38∶3)
828.56	0.11	a	PC(P-40∶1)
832.56	0.16	a	PC(O-40∶1)
834.64	0.04	a	PC(40∶6)

All [M+H]^+^ except 449.33 and 489.27, which correspond to [M+K]^+^ and [M+Na]+ respectively. #Theoretical identity (not confirmed by MS^n^).

**Figure 7:**
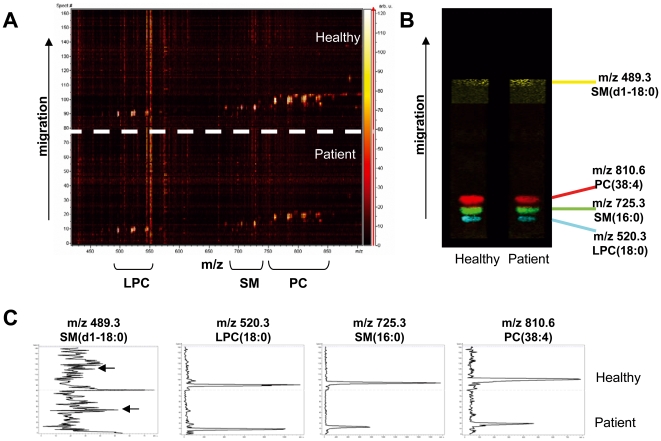
TLC-MALDI analysis of raw extracts from one CF patient and one healthy control. A: 2D diagram representing m/z (horizontal axis) and chromatographic separation (vertical axis) from patient and control samples. In both cases extraction was performed from 200 µl of plasma, and an equal volume of extract was spotted on the TLC plate. B: 1D diagram representing the chromatographic separation on the TLC plate. Colors correspond to individually selected m/z values by the FlexImaging software. C: Semiquantitative chromatographic integration by the TLC-MALDI software, corresponding to the peaks selected in B from healthy control and patient samples. Arrows indicate the peaks corresponding to m/z 489.3 according to the chromatographic migration.

In order to illustrate the potential of our findings in the screening of individuals, and given that the results concerned phospholipid signatures, we chose a new technique (TLC-MALDI) especially adapted to phospholipid analysis [21]. We analyzed the raw extracts from one healthy control and one patient (none of them included in the original study) to TLC-MALDI. Figure 7 shows 1D and 2D TLC-MALDI plots corresponding to both individuals. As it can be observed in Figure 7A, LPC, SM and PC signatures present generally stronger intensity in the control. The color identification of selected peaks on the TLC plate by the FlexImaging software (Fig. 7B) reveals a stronger signal for LPC(18∶0), SM(16∶0) and PC(38∶4) in the control sample, and a weaker signal for SM(d18∶0), which is consistent with the results obtained in the study. This can be visualized by the chromatographic integration performed by the TLC-MALDI software (Fig. 7C). This is particularly clear in chromatograms for SM(16∶0) and PC(38∶4). However, it must be considered that these results are not quantitative, though they suggest a similar trend as those obtained by ClinProTools™. This analysis represents the first application of TLC-MALDI to human plasma samples, as well as a complement to other MS methods used for identification of lipid structures.

## Discussion

In this study, we found some significant changes in the plasma content of several phospholipids species in healthy *vs* all CF individuals, and in mild *vs* severe CF patients. This includes a significant decrease in a number of LPC and PC species in all CF patients. This finding, confirmed by two independent statistical approaches, is consistent with the majority of reports on lipid alterations associated with CF that have been published to date. However, two major points make these results novel and unique: one technical and one physiopathological.

From the technical point of view, in this study we have used a novel approach that reveals promising for further studies aiming at the search for phenotypical markers associated with disease severity and prognosis. It can be argued that the results obtained correspond to some of the most abundant lipid species in biological samples. In fact, for this first study we have chosen a standard matrix, like DHB. This favors the detection in the positive mode, and in particular that of PC species as compared to other phospholipids. Nevertheless, we have been able to find signatures corresponding to neutral lipids and free fatty acids, and they were found statistically unchanged. On the other hand, the purification method employed did not allow the analysis of less abundant acidic phospholipids.

The search for prognostic lipid biomarkers of disease severity and exacerbations should allow a better understanding of the regulation of inflammation, the link between genotype and lipid imbalances and the therapeutic potential of certain lipids. In this report, we propose to apply a technique initially developed for the exploration of protein biomarkers in the quest for lipid biomarkers. Since it is based on MALDI technology, it is a fast and user-friendly methodology, and compatible with several previous extraction and separation approaches (i.e., liquid and column chromatography). Yet, even though the statistical analyses provided by ClinProTools™ are robust, our results also suggest the convenience of performing powerful statistical analyses in parallel in order to validate the results obtained.

We also present TLC-MALDI as a complementary method especially suitable for sample screening and for comparison of lipid signatures from human fluids. In fact, TLC-MALDI gathers a number of advantages. Firstly, it comprises a separation method different from liquid or column chromatography, and more reproducible, as several samples can be run in parallel exactly in the same conditions. Secondly, the procedure has the rapidness and user-friendliness of MALDI.

This semiquantitative method allows an “at a glance” way to visualize the plasma lipidome of an individual at a particular time and condition. It could be considered as the lipidomic equivalent to the western blot in proteomics. In this sense, it represents an ideal approach to detect evolving changes as a function of severity, exacerbations of disease, and therapeutical interventions. It can be a method of choice when the number of samples is limited, or to verify the results obtained after a statistical analysis on samples that have not been included in the analysis (as in this case) or chosen randomly from the analysis group. The present report is, to our knowledge, the first application of TLC-MALDI to lipid screening in human plasma.

The next step in the search for prognostic lipid signatures in plasma is to follow up patients during the development of the disease and to compare the signatures from those who remain with a mild phenotype with those who evolved to a severe one. This long term prospective study needs a strong participation of a clinical team, and a precise and standardized definition of mild and severe phenotypes, which should not change during the course of the study. Unfortunately, this is not the case at present. For example, the definition of exacerbation is still a matter of discussion among CF clinicians. Of note, our definition of severity *vs*. mild is clearly described in the Methods section, and represents a consensus between physicians from Poland, Sweden, and France, as reached during our FP6 CE project (NEUPROCF).

Physiopathologically, we have found a significant correlation between some of the decreased molecules and the severity status of patients concerning respiratory symptoms. This includes 5 PC forms containing highly unsaturated chains. This is in agreement with the lower polyunsaturated fatty acid levels found in blood and tissues from patients and models. Linoleic acid (18∶2n−6) has been consistently found decreased in phospholipids, triacylglycerides and cholesteryl esters in plasma and serum from CF patients [28], [29]. Docosahexaenoic acid (22∶6n−3) has been found decreased in tissue phospholipids from cftr^−/−^ null mice [15], while arachidonic acid (20∶4n−6) is decreased in plasma and tissue phospholipids from the cftrtm1HGU/tm1HGU mouse [30]. Nevertheless, our results cannot be compared directly with those of previous studies on fatty acid profiling of CF patients, as they respond to totally different approaches: analysis of intact molecules in the present study and of cleaved fatty acid moieties in the latter.

Our observations can be roughly summarized as a consistent decrease in several PC and LPC species in CF plasma. Other authors have found similar results [31]. This can be attributed to (i) maldigestion-malabsorption, (ii) abnormal metabolism in liver, (iii) alterations in plasma lipoproteins or apolipoproteins, (iv) increased degradation or turnover, or a combination of them.

As recently reported, lysophospholipids and phospholipids are excreted at a higher extent in CF patients [32]. This is attributed by the authors to a defect in fat absorption and choline depletion. However, they do not find any differences in total phospholipid, PC or LPC content in blood, which could be due to a defect in bile function and is found concomitant with an increase in phosphatidylethanolamine conversion to PC in the liver. To this regard, it is worth noting that in our study no significant differences are found between pancreatic sufficient and pancreatic insufficient patients, in agreement with other reports [14], [33]. This challenges the hypothesis of an exclusive maldigestion-malabsorption origin.

If phospholipid metabolism is concerned, an important pathway to consider is the methionine-homocysteine cycle, in which choline *via* betaine provides methyl groups to regenerate S-adenosylmethionine, an important element in the generation of PC and amino acid precursors for glutathione [34], [35]. Liver triacylglycerol accumulation and oxidative stress are common in CF and also occur in choline deficiency [36]. Reduced ratios of S-adenosylmethionine to S-adenosylhomocysteine and of PC to phosphatidylethanolamine in plasma, and phospholipid malabsorption have also been reported in CF children [36]. These altered values are reversed by PC treatment [36] and also by administration of 5-methyltetrahydrofolate [37]. Another enzyme involved in PC synthesis is CTP∶phosphocholine cytidylyltransferase. A recent work reports its decreased expression in *P.aeruginosa*-infected murine CF lung epithelium [38]. However, the fact that one potential sphingomyelin form, coincident in mass with the sodium adduct of SM(d18∶0) (sphinganine-1-phosphocholine or lysosphingomyelin), which also contains the phosphocholine head group, is significantly increased in CF patients, represents an exception to the hypothesis of an alteration in choline metabolism and/or availability.

The finding of a sphingolipid species increased in the plasma of all CF patients *vs* healthy individuals recalls the recently reported accumulation of ceramides in *Cftr*-deficent cells and the therapeutic potential of the acid sphingomyelinase inhibitor amitriptilin [39]. Whether there is a link between the membrane ceramide and the presence of a sphinganine-containing lysosphingomyelin in plasma may represent an interesting field for future research. Sphinganine is the saturated form of sphingosine, both sphingoid base components of sphingomyelins. Sphinganine-containing molecules are elevated in malignant tumors as compared to normal tissues [40], and a higher content can be attributed to a decrease in dihydroceramide desaturase activity. However, no information is available on plasma levels of these compounds. Likewise, nothing is known about the ceramide content in plasma of CF patients.

Concerning the alterations observed in LPC, our findings could be attributed to the presence of spontaneously degraded forms of PC moieties. However, no differences were found between mild and severe patients. If LPC and PC alterations have independent origins, a potential hypothesis for increased LPC is a higher activity of lecithin-cholesterol acyl transferase (LCAT), which has been reported in CF patients [41]. It must be noted that decreased levels of unsaturated forms of LPC (containing 18∶2n−6 or 18∶1n−9 chains) have been described as markers of colorectal cancer [42]. If, on the contrary, LPC and PC alterations have the same origin, this could involve either malabsorption or a defective transport by lipoproteins or both.

To this regard, the fatty acid pool is decreased in plasma lipoproteins from CF patients [43]. LDL and HDL are decreased in serum from CF patients, along with apoB, apoA1 and cholesterol, while VLDL and triglycerides are augmented, according to a pioneering study [44]. A decreased synthesis of apoB and apoA-I in intestinal epithelium from CF patients has recently been confirmed [45], suggesting a direct effect of CFTR dysfunction on malabsorption and/or abnormal formation of lipoproteins.

Finally, plasma depletion in PC species could be due to an increased utilization by cells. In CF fibroblasts and platelets, an increased incorporation of choline into membranes has been observed, in parallel to an unchanged PC content, suggesting an increased PC turnover in these cells [46].

As a consequence of the lower PC concentration in plasma, its availability for other tissues can be compromised. PC alterations in CF have also been reported in lung, where it constitutes the main component of surfactant. In this context, PC, along with other surface-active phospholipids, has been found decreased in tracheobronchial secretions from CF patients [47], [48].

PC depletion may also reflect or have profound consequences on cell membrane composition and architecture including that of membrane micordomains. It has been demonstrated that CFTR recruitment in detergent resistant micordomains participates in the regulation of the cell response to infection and inflammation [49], [50]. Assuming that the PC and SM content in micordomains is changed, one can postulate that the microdomain-related recruitment of CFTR may be modified. In addition, phospholipids bind to cytoskeletal proteins, and as such they may influence the trafficking of membrane proteins. It has been observed, for example, that the NBD1 domain of CFTR binds selectively to phosphatidylserine, and that its mutated form (F508del), loses this ability. This selective binding may play a role in the delivery of this protein to the plasma membrane [51]. In both cases, a treatment aimed to modify the phospholipid content of cells may represent a way to correct the cell function in CF.

In conclusion, in addition to a general decrease in LPC and PC species, as well as an increase in SM(d18∶0) in the plasma from CF *vs* healthy individuals, we show decreased levels of PC(36∶3), PC(36∶5), PC(38∶6), and PC(38∶5) in plasma as a potential signature of respiratory disease severity in CF. This constitutes the first application of MALDI-TOF-ClinProTools™ and of TLC-MALDI to lipidomic analysis of human-derived samples, and an alternative approach for blood lipidomics in CF.
